# Complete genome sequence of *Clostridium beijerinckii* DSM 105335 obtained by hybrid Illumina- and Nanopore-based assembly

**DOI:** 10.1128/mra.01445-25

**Published:** 2026-08-03

**Authors:** Jessica M. O'Loughlin, Emma Allen-Vercoe, Catherine McNay, Sarah Windsor, Louise Horsfall, Karl Burgess

**Affiliations:** 1 School of Biological Sciences, The University of Edinburgh 98275, Edinburgh, United Kingdom; 2 Department of Molecular and Cellular Biology, University of Guelph 317113, Guelph, Ontario, Canada; 3 FUJIFILM Biotechnologies, Billingham, United Kingdom; 4 FUJIFILM Biotechnologies, Raleigh, North Carolina, USA; University of Maryland Baltimore, Baltimore, Maryland, USA

**Keywords:** enteric bacteria, gram-positive bacteria, veterinary microbiology, fermentation, genomes, DNA sequencing, anaerobes

## Abstract

*Clostridium beijerinckii* DSM 105335 was originally isolated from wild boar feces in Canada. Here, we report a complete 5,792,971 bp genome sequence for this strain. It was assembled using a hybrid approach utilizing short Illumina paired-end reads and long reads sequenced with an Oxford Nanopore MinION.

## ANNOUNCEMENT

*Clostridium* spp. are anaerobic, spore-forming, and gram-positive bacteria commonly found in the soil (1, 2) and gastrointestinal tracts (3, 4) and can be used in industrial bioprocesses (5–8). To increase the available genomic information for these strains, we report the complete genome sequence for *C. beijerinckii* DSM 105335, which was originally isolated from wild boar feces in Stratford, Ontario (Canada), collected within 10 min of defecation and stored at −80°C. A “core” region from the sample was cultured in nutrient agar (DSMZ medium 1) in an anaerobic atmosphere at 37°C (9).

The lyophilized version of this isolate was ordered from DSMZ and revived in DSMZ 104 media following the anaerobic bacteria DSMZ protocols (10, 11). The sequenced isolate had a single colony inoculated into DSMZ 104 media and grown to exponential phase (anaerobic atmosphere, N_2_/H_2_/CO_2_, 37°C). The bacteria were PBS washed, resuspended in DNA/RNA shield inactivation buffer (Zymo Research, USA), lysed using Tris-EDTA (TE) buffer containing lysozyme (MPBio, USA), metapolyzyme (Sigma-Aldrich, USA), and RNase A (ITW Reagents, Spain), and treated with proteinase K (VWR Chemicals, Ohio, USA) and SDS (Sigma-Aldrich, Missouri, USA). The genomic DNA was SPRI bead-purified and resuspended and diluted in EB buffer.

Illuminia libraries were prepared (Nextera XT Library Prep Kit, Illumina, San Diego, USA) following the manufacturer’s protocol, except: input DNA was increased two-fold, and a 45 s PCR elongation time was used. Library preparation was performed via transposon-based DNA fragmentation and tagging as per kit instructions. The libraries were sequenced (Illumina NovaSeq 6000, Illumina, San Diego, USA) using a 250 bp paired-end-protocol. Each software package’s default parameters were used unless otherwise specified. Reads were adapter-trimmed (Trimmomatic-0.30 [12], sliding window quality cutoff Q15). The quality of the trimmed reads was assessed using MicrobeNG’s housescripts with Samtools-1.23 (13), BedTools-2 (14), and BWA-MEM-0.7.19 (15) software. The Illumina assembly statistics were calculated using QUAST (16) (Table 1).

**TABLE 1:** Summary of sequencing and hybrid assembly key statistics and results

Stage	Statistic	Value
Illumina sequencing	Number of reads (before trimming)	2,681,795
	Number of reads (after trimming)	2,665,086
	Median insert size	585
	*N*_50_	107,916
Nanopore sequencing	Number of reads	109,540
	Mean read length	16,087.0
	*N*_50_	27,096.0
Hybrid genome assembly	Number of contigs	1
	Length of contig	5,792,971
Genome annotation	Total number of genes annotated	5,232
	Total number of coding DNA sequences (CDSs)	5,084
	Total coverage of annotated genes on the genome	81%

For long-read sequencing, DNA libraries were prepared for an Oxford Nanopore Technologies (ONT) GridION (SQK-NBD114.96 or SQK-RBK114.96 Kit, ONT, United Kingdom). Shearing was not performed for this workflow. Barcoded samples were pooled together, loaded in a FLO-PRO114M or FLO-MIN114 (R.10.4.1) flow cell, and basecalled (Dorado-7.4.14 [17]). Hybrid assembly was performed (Unicycler-0.4.0 [18]), and contigs annotated (National Center for Biotechnology Information [NCBI] Prokaryotic Genome Annotation Pipeline 6.10 [19]) (Table 1). The final assembled genome had a circular topology that rotated to the chromosomal replication initiator protein Dna A (mapped in Bandage-0.8.1 [20 using BLAST+ 2.17.0) (21).

Its assembly size (5,792,971 bp) and 29.67% GC content are in line with the expected GC content (29.8%) and genome size (6,220,000 bp) for *C. beijerinckii*. The 16S sequence (identified using Barrnap-0.9 (22) was matched in the SILVA 16S database (23) (release 128) with 100% identity to the *C. beijerinckii* to the species level, suggesting high quality sequencing and confident species identification.

## Data Availability

This genome project for *C. beijerinckii* DSM 105335 is recorded under the BioProject PRJNA1320638, with its genome sequence (CP199770) and its 16S rRNA sequence (PX651966) at GenBank. The Illumina reads and Nanopore data are available under SRA SRR35569608 and SRR35569606, respectively. The log and graph files (.gfa) generated with Unicycler for this assembled genome are available at Figshare (https://doi.org/10.6084/m9.figshare.30187555.v1).
